# Impact of the Z potential technique on reducing the sperm DNA fragmentation index, fertilization rate and embryo development

**DOI:** 10.5935/1518-0557.20170055

**Published:** 2017

**Authors:** Carlos Duarte, Víctor Núñez, Yat Wong, Carlos Vivar, Elder Benites, Urso Rodriguez, Carlos Vergara, Jorge Ponce

**Affiliations:** 1 NIU VIDA Specialized Center for Assisted Reproduction, Lima, Peru; 2 Laboratory of Animal Biotechnology, Ricardo Palma University, Lima, Peru

**Keywords:** Z potential, sperm DNA fragmentation, embryo development

## Abstract

**Background:**

In assisted reproduction procedures, we need to develop and enhance new protocols to optimize sperm selection. The aim of this study is to evaluate the ability of the Z potential technique to select sperm with intact DNA in non-normospermic patients and evaluate the impact of this selection on embryonic development.

**Methods:**

We analyzed a total of 174 human seminal samples with at least one altered parameter. We measured basal, post density gradients, and post density gradients + Z potential DNA fragmentation index. To evaluate the impact of this technique on embryo development, 54 cases were selected. The embryo development parameters evaluated were fertilization rate, cleavage rate, top quality embryos at the third day and blastocysts rate.

**Results:**

We found significant differences in the study groups when we compared the sperm fragmentation index by adding the Z potential technique to density gradient selection vs. density gradients alone. Furthermore, there was no significant difference in the embryo development parameters between the low sperm fragmentation index group vs. the moderate and high sperm fragmentation index groups, when selecting sperms with this new technique.

**Conclusions:**

The Z potential technique is a very useful tool for sperm selection; it significantly reduces the DNA fragmentation index and improves the parameters of embryo development. This technique could be considered routine for its simplicity and low cost.

## INTRODUCTION

Primary infertility is mainly defined as the incapacity of a couple to conceive within 12 months of periodic sexual intercourse without any contraceptive method; this condition affects approximately 15% of couples in reproductive age and its prevalence is increasing (Evers, 2002; Zegers-Hochschild *et al.*, 2009).

In approximately 50% of these cases, the underlying problem lies in the male factor, a multifactor syndrome that encompasses a wide variety of disorders (Varghese *et al*., 2008; O'Flynn O'Brien *et al*., 2010). These disorders can be caused by intrinsic as well as environmental factors. In the latter group, one of the main causes is the high production of reactive oxygen species, that impact the spermatogenesis process, triggering low sperm production, mitochondrial problems, morphological aberrations, spermatozoa with fragmented DNA, and an active apoptosis pathway (Tahmasbpour *et al*., 2014; Wallach *et al*., 2010; Punab *et al*., 2017).

Seminal preparation techniques routinely used for assisted reproduction treatments are density gradient centrifugation (DGC) and swim-up. These techniques focus their methodology on the basics of sedimentation or migration to separate the best spermatozoa, and they are both efficient in selecting morphologically normal sperms with a high degree of nuclear maturity (Le Lannou & Blanchard, 1988). However, other important sperm characteristics such as DNA integrity, apoptosis, membrane maturation and ultrastructure are not directly addressed by these techniques, even more so when we start from a sample showing asthenozoospermia and/or teratozoospermia. Therefore, these techniques do not guarantee the genomic integrity of the selected spermatozoa (Marchesi *et al.*, 2010).

Up to date, different sperm selection methodologies have been developed to improve conventional seminal preparation protocols for assisted reproduction procedures (Beydola *et al*., 2013; Henkel, 2012). These methods aim to select mature sperms, structurally appropriate, non-apoptotic and with a high DNA integrity index. According to the strategies used in its methodology we can find: (I) surface charge (electrophoresis and Z potential), (II) apoptosis (selection of spermatozoa by filtration in magnetic columns, glass fiber columns), (III) sperm membrane maturity (hyaluronic acid binding) and (IV) ultra-morphology (IMSI) (Yetunde & Vasiliki, 2013).

The Z potential method (ZP) combines DGC and a selection based on the sperm membrane electric potential to obtain sperms with a negative charge (-16mV to -22 mV), this is evidence for proper epididymal maturation and spermatogenesis at a testicular level. (Giuliani *et al*., 2004). These selected spermatozoa show important favorable changes such as: proper nuclear compaction, exchange of nuclear proteins and CD 52 glycopeptide buildup, rich in sialic acid residues; this last characteristic of the sperm maturation process is the main one responsible for the negative value charge in the sperm plasmatic membrane. Concurrently, this loading value enables a better *in vivo* interaction with the oocyte membrane and avoids non-specific aggregations in the female tract, as well as among the spermatozoa themselves (Ishijima *et al*., 1991; Smith *et al*., 2015).

It has been described that the Z potential technique significantly improves total motility, progressive motility, morphology index, hyper activation level, DNA integrity and maturity, percentage of sperms without protamine deficiencies and selects better sperms without the active apoptotic pathway (Chan *et al*., 2006; Zarei-Kheirabadi *et al*., 2012).

Nasr Esfahani *et al*. (2016) showed that the Z method improves the percentage of good-quality embryos and pregnancy rates, when it is compared to the DGC technique. They also indicate a favorable increase in the selection of female sex in the reported births.

However, further clinical trials are still necessary to evaluate this technique potential over routine and complex techniques already used in the clinical field. Therefore, the aim of this study was to evaluate the ability of the Z potential technique to reduce levels of DNA fragmentation during the seminal preparation process in non-normospermic patients, and evaluate the impact of such selection on embryo development parameters.

## MATERIALS AND METHODS

### Patient selection and experimental design

This prospective study was carried out at the Niu Vida Specialized Center for Assisted Reproduction (Lima, Peru), between October 2016 and February 2017, Niu Vida's Ethics Committee approved this study.

To analyze the Z Potential technique effect on reducing the sperm fragmentation index in men with low, moderate and high DNA Fragmentation Index (DFI). 174 seminal samples were included, with at least one altered parameter according to World Health Organization (WHO, 2010). Samples were randomly selected using the algorithm provided by RANDOM.ORG and were distributed at our own criteria in three groups according to their basal DFI as follows: Group A, low DFI (0% - 16%); Group B, moderate DFI (16% -26%) and Group C, high DFI (≥27%).

We compared the DFI reduction percentage in the post-DGC and post-DGC + ZP populations independently by the Student's t-test for related samples with a *p*<0.05 as discriminatory value. Data normality was verified by the Kolmogorov-Smirnov (KS) test, and the equality of variances with the Levene test. We analyzed the difference of DFI between DGC vs. DGC + PZ between groups using ANOVA and Games Howell's Post Hoc analysis.

To evaluate the ZP technique impact of on embryo development parameters, we choose 54 randomized high complexity cases with 519 donated oocytes using the algorithm provided by RANDOM.ORG. Selected cases were assigned two groups, using DFI as selection criteria, as follows: Group 1, DFI ≤16%, 298 oocytes, and Group 2, DFI ≥17%, 221 oocytes (Avendaño *et al.*, 2010). The parameters evaluated were: fertilization rate (FR), cleavage rate (CR), good quality embryos at the third day (TQE) and rate of blastocysts (BR). The data normality was analyzed using Kolmogorov-Smirnov (KS) test, and the results of each parameter were evaluated using the Mann Whitney U test.

### Seminal sample preparation through density gradient columns

The samples were obtained by masturbation with abstinence of 3-5 days, and classical seminal parameters were measured according to the WHO (2010).

After corroborating sample liquefaction, a 25µl aliquot was taken to evaluate basal DFI, after that, the samples were placed on a density gradient column formed by an upper layer of 45% and a bottom layer of 80% (SupraSperm (Origio) + Flushing Medium (Origio)). The tubes were centrifuged at 1000rpm for 10 minutes. Sperm pellets were recovered and washed in flushing medium (Origio) at 1000rpm for 10 minutes and finally reduced to a volume of 400µl - a 25µl aliquot was separated to evaluate the DFI.

### Sperm selection by zeta potential

Sperm selection by Z Potential was carried out modifying the Chan *et al.* (2006) protocol. Immediately after the DGC procedure, the reduced 400µl volume was exposed to a positive charge on the walls of a 15ml Falcon polystyrene tube - this positive charge was previously induced by rubbing the tube within a latex glove for 5 consecutive turns. The tube was left to stabilize with the sample for 1 minute at room temperature (RT), to favor highly electronegative spermatozoa adhesion. All the medium was removed by aspiration using a 3mL-polyethylene Falcon transfer pipet, the adhered sperms were finally recovered by washing the walls with 500 ul of 3% Human Serum Albumin (HSA), diluted in flushing medium (Origio).

### Sperm DNA fragmentation index

Each sample DNA Fragmentation Index (DFI) was measured at 3 different times (basal, post DGC and post DGC + PZ), using the Spermatic Chromatic Dispersion (SCD) technique with the Halosperm^®^ commercial kit, following the manufacturer's instructions. 500 sperms were counted per sample, and the DFI was calculated by dividing the number of spermatozoa with small or nil halo by the total number of analyzed spermatozoa multiplied by 100.

### Ovarian stimulation

For all cases, a single stimulation protocol was carried out using rFSH (Elonva^®^) on day 3 of the menstrual cycle (MC), rFSH (Gonal^®^) from day 10 to day 12 of MC, cetrorelix acetate (Cetrotide^®^) from day 10 to day 12 and triptorelin (Gonapeptyl^®^) at day 14 of MC. Oocyte capture was performed by vaginal ultrasound under general anesthesia with Propofol (Diprivan^®^ 1% P/V) in Global^®^ Total^®^ w/Hepes medium.

### Intracytoplasmic sperm injection

The oocytes collected were pre-incubated in Global^®^ Total^®^ medium for fertilization for 2 hours, then they were mechanically denuded by a brief exposure to 80 IU of hyaluronidase. All oocytes included in the study were in metaphase II and any morphological abnormality was considered exclusion criteria. The spermatozoa were injected prior to immobilization in PVP on an inverted Olympus IX70 microscope, equipped with Research Instruments^®^ micromanipulators and microinjectors.

The FR was obtained by dividing the number of fertilized oocytes (2PN) among the total number of injected oocytes, multiplied by 100, the CR was obtained by dividing the number of cleaved embryos by the number of fertilized embryos, multiplied by 100. Good-quality embryos in day 3 were defined as those that had between 6 and 8 cells, with blastomeres of equal size, and a fragmentation rate below 25%. Day 3 TQE was obtained by dividing good quality embryos between the number of cleaved embryos multiplied by 100. BR was obtained by dividing the number of Blastocysts obtained on day 5 by the total number of fertilized embryos, multiplied by 100.

## RESULTS

### Z potential and DFI reduction

Table 1 presents descriptive values and standard deviations of the 3 groups. Group A had an average basal DFI of 10.2%. Group B had an average basal DFI of 20.4% and Group C had an average basal DFI of 33%. DFI average values by DGC were 4.1%, 9.2% and 15%, whereas DFI average values by DGC + PZ were 1.2%, 3.1% and 5% for Groups A, B and C respectively.

**Table 1:** Average sperm DNA fragmentation index.

	Group A (DFI 1% - 16%) (n = 88)	Group B (DFI 17- 26%) (n = 59)	Group C (DFI ≥ 27%) (n = 27)
Basal DFI (%)	10.2±4.1	20.4±2.4	33±1.4
Post DGC DFI (%)	4.1±1.3	9.2±1.2	15±2.4
Post DGC+ PZ DFI (%)	1.2±0.3	3.1±1.7	5±2.3

To compare the DFI reduction between DGC and DGC + PZ techniques in Groups A, B and C, the Student's T-test was performed for independent samples in each population. The results depicted on Table 2 show that the DGC + PZ technique has an DFI reduction value of 83.2, 83.94 and 83.48 in each group, respectively; and that there is a significant difference (*p*<0.05) with the DGC technique.

**Table 2:** DFI reduction comparison between DGC and DGC + PZ.

	Grupo A (n=88)	Grupo B (n=59)	Grupo C (n=27)
DFI Red. DGC (%)	63.91±5.7	57.48±9.4	53.82±13.99
DFI Red. DGC + PZ (%)	83.2±3.1*	83.94±7.4*	83.48±8.1*

* Indicates significant difference where *p*<0.05.

To determine in which group there is a greater reduction of the DFI by the DGC + PZ technique, we analyzed the difference between the reduction values in each group. Table 3 shows that there is a significant difference between Groups B and C with respect to Group A (29.6% and 26.24% vs. 19%), there was no difference between Groups B and C.

**Table 3:** Comparison of the DFI reduction between DGC and DGC + PZ.

Comparison of the DFI reduction between DGC and DGC + PZ	
Groups	∆ Red. DFI (%)
Group A (n=88)	19.31±15.23^a^
Group B (n=59)	26.24±9.9^a^^,^^b^
Group C (n=27)	29.66±9.05^a^^,^^b^

a Indicates significant difference where *p*<0.05;

b Indicates no significant difference where *p*>0.05.

### Z potential and embryo development

The effects of the Z potential on embryo development was analyzed forming 2 groups: Group 1 with basal DFI ≤16 and Group 2 with basal DFI ≥17. Table 4 shows the descriptive values for each parameter studied. The Mann Whitney U analysis was performed, and we found that there were no statistical differences between the Groups 1 and 2 for the embryo development parameters studied (FR *p*=0.82, CR *p*=0.97, TQE *p*=0.29, BR *p*=0.096).

**Table 4:** Embryo development parameters.

	Group 1	Group 2	*p* value
Fertilization Rate (%FR)	80.31±4.01	78.73±4.40	0.82
Cleavage Rate (%CR)	89.52±4.58	89.44±3.38	0.97
Top Quality Embryos (%TQE)	83.55±9.68	90.07±2.40	0.29
Blastocyst Rate (%BR)	58.48±4.08	61.68±4.48	0.096

## DISCUSSION

The DGC technique results in a sperm homogeneous population with better classical parameters, and reduces DFI significantly (Torabi *et al.*, 2017; Zhao *et al.*, 2016); however, it presents limitations in severe pathologies and high DFI (Zini *et al.*, 2000). DFI values in B and C groups (9.2% and 15%) are quite close to those found for Wang *et al.* (2014), who reported a DFI value of 19% in astenozoospermic samples and 16% DFI in oligozoospermic samples, confirming our results - the limitations of this technique.

Our analysis of DFI reduction post DGC and post DGC + ZP showed significant differences in each group. This reduction (Table 2) shows an improvement in respect to the classic density gradient method, regardless of the degree of the sperm sample basal DFI, indicating that implementing the ZP technique to DGC improves sperm selection in terms of DNA integrity, this results are consistent with those reported by Khajavi *et al.* (2009).

At the same time, our results show that the largest decrease in DFI occurs in populations with medium and high degrees of fragmentation (Group 2 and Group 3) (Table 3). This trend demonstrates the technique's performance in retrieving the largest amount of mature sperms, both at the level of chromatin structure and at the sperm membrane with a low apoptosis level (Zarei-Kheirabadi *et al*., 2012).

Negative effects of sperm DNA fragmentation over IVF or ICSI treatments has been extensively evaluated and demonstrated by Simon *et al.* (2017) meta-analysis, which includes 8068 cases. At the same time, Avendaño *et al.* (2010) reported a 16% DFI cut-off value, from this value there is a negative impact on the pregnancy rate per embryo transfer. No significant differences were found (*p*<0.05) in each comparison between groups 1 and 2 (DFI ≤16, DFI >16 respectively) for each analyzed parameter (Table 4). These findings confirm that the Z potential technique is efficient and independent of the DFI basal value.

Considering only group 2 (DFI >16%) as having clinical relevance, we found that our percentage of fertilization value (78.7%) is comparable to that reported by Nasr Esfahani *et al.* (2016) (79%), employing sperm hyaluronic acid binding (PICSI) in altered male factor cases, and it is higher than the value reported by Dirican (2008) (70%), employing DGC + MACS technique in men with oligozoospermia, teratozoospermia and astenozoospermia (Dirican *et al.*, 2008); and it is also higher than the value reported by Gianaroli *et al.* (2008) (74%), and Gianaroli *et al.* (2010) (69%) employing polarized light for sperm selection.

Our analysis of our CR value (89.4%) in the group of clinical relevance (Group 2) showed that it was in accordance with the one reported by Kheirollahi-Kouhestani *et al*. (2009), who published a 92.24% CR value when they also employed the Z potential technique; however, this study did not report on the other parameters studied by our group. García-Ferreyra *et al.* (2014), employing MACS, reported a CR of 98.3%, TQE on day 3 of 88.4% and a BR of 50.8%. In comparison with our group the CR was 89%, a lower value; however, TQE on day 3 was comparable (90.07%) and our BR (61.68%) was greater.

Nasr Esfahani *et al.* (2016), employing the Z potential technique, reported an FR value of 67.81% and a TQE in day 3 value of 45.83%. These results are similar to ours, with an FR value of 78.7%; however, his rate of TQE on day 3 is lower, which could be explained by the age of the patients in their study. It is important to highlight that Nasr Esfahani *et al.* (2016) reaffirms what we found in andrology research, since all the parameters of embryo development studied showed significant difference compared to when only DGC is used in sperm selection.

## CONCLUSIONS

Our results shown that the Z potential technique, *per se* is a very useful tool for sperm selection in assisted reproduction treatments; it significantly reduces the sperm DNA fragmentation index, regardless of the sample initial basal value, and improves the parameters of embryo development achieved with conventional techniques for seminal preparation.

Hence, the Z potential technique is an option that could be considered routine due to several advantages in respect to other techniques (DGC, MACs, PICSI, IMSI, etc.), stressing its simplicity, processing velocity and low-cost of implementation and start-up.

This new tool could even replace a previous diagnosis of sperm DNA fragmentation index when couples have already opted for an IVF treatment, which would reduce costs for patients by giving them a better chance to success in their treatments, comparable to when more complex and more expensive techniques are used for sperm selection.
